# Supplementary figures and images for: NCOA3 identified as a new candidate to explain autosomal dominant progressive hearing loss
Source: Hum Mol Genet. 2020 Dec 17;29(22):3691–705. doi: 10.1093/hmg/ddaa240 (PMC7823111; doi:10.1093/hmg/ddaa240)

Supplementary Figure 1

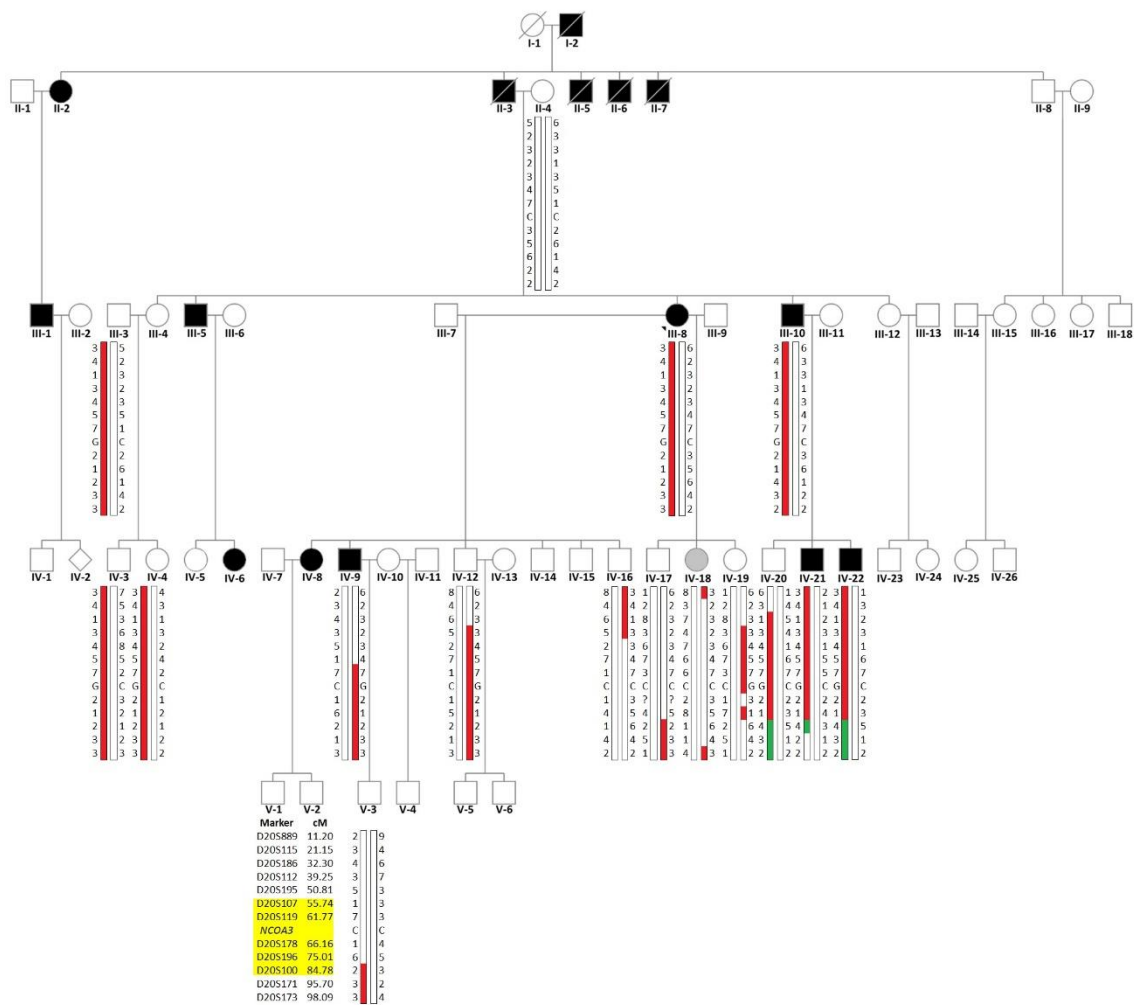

Supplement: Sup_Fig_1_ddaa240 [file sup_fig_1_ddaa240.pdf]

Supplementary Figure 2

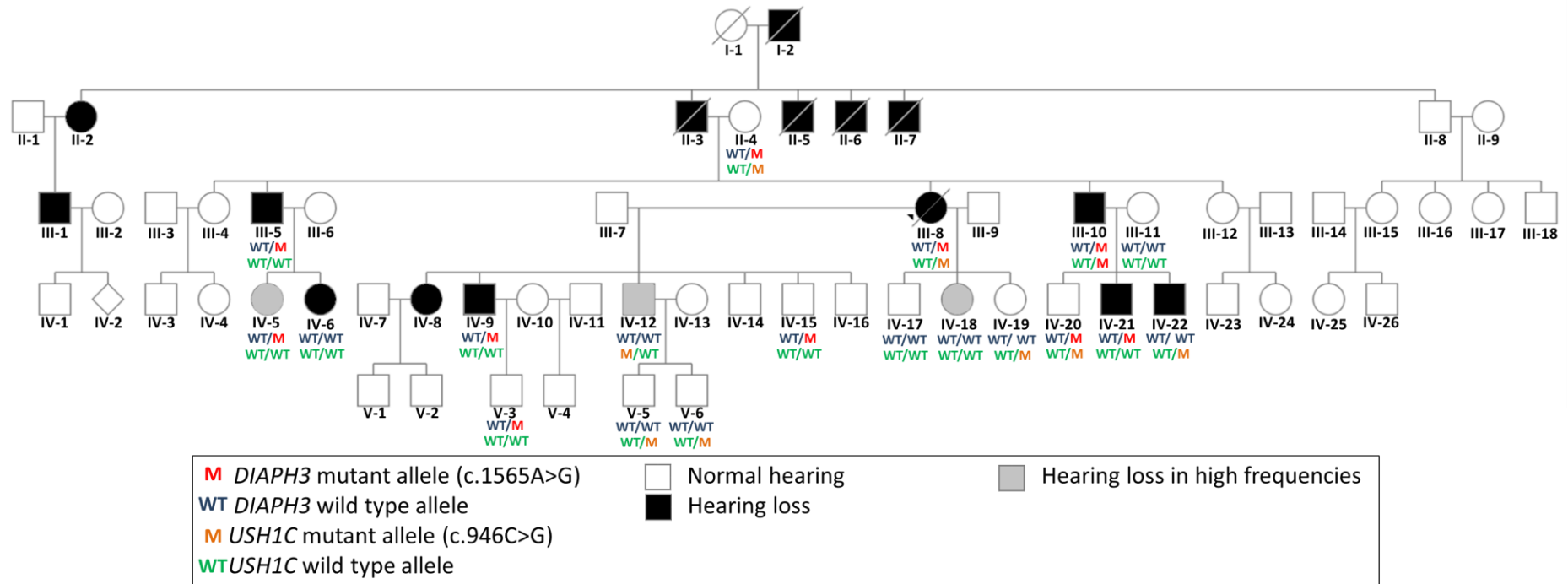

Supplement: Sup_Fig_2_ddaa240 [file sup_fig_2_ddaa240.pdf]

Supplementary Figure 3

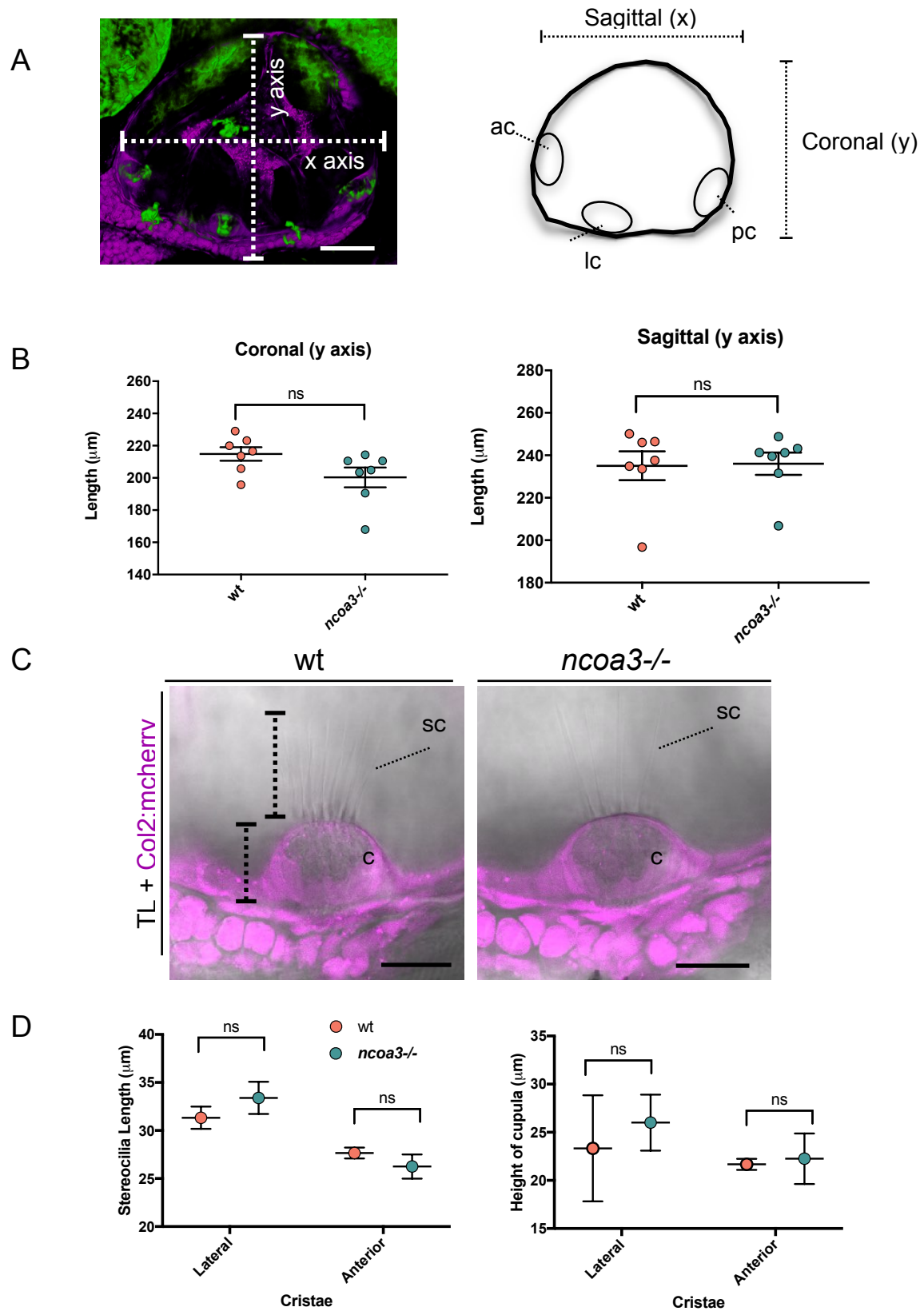

Supplement: Sup_Fig_3_ddaa240 [file sup_fig_3_ddaa240.pdf]

Supplementary Figure 4

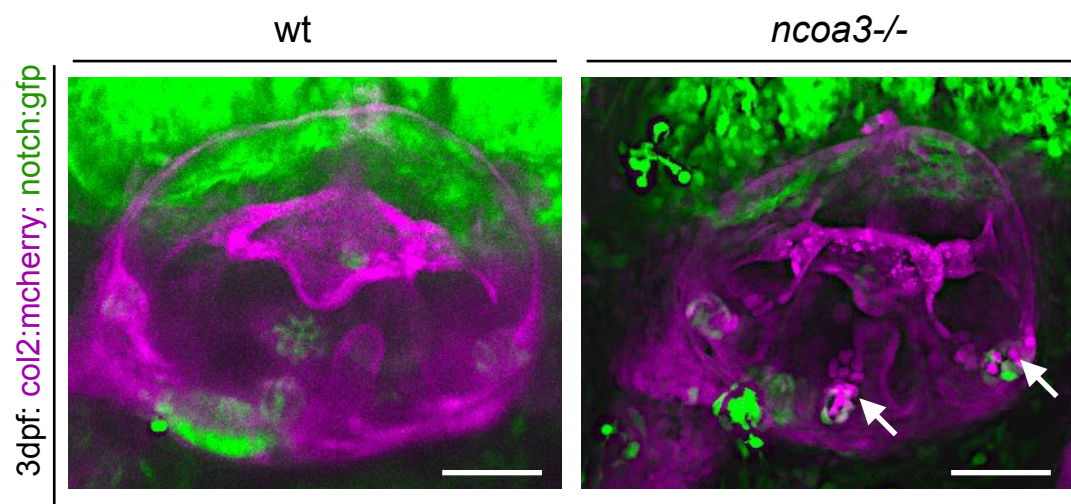

Supplement: Sup_Fig_4_ddaa240 [file sup_fig_4_ddaa240.pdf]
